# A longitudinal study of early childhood caries incidence in Wenzhou preschool children

**DOI:** 10.1186/s12903-017-0394-1

**Published:** 2017-07-04

**Authors:** Xiping Wang, Zhiyuan Wei, Qiao Li, Liqin Mei

**Affiliations:** Department of Preventive and Pediatric Dentistry, School and Hospital of Stomatology, Wenzhou Medical University, Wenzhou, 325027 China

**Keywords:** Children, Cohort study, Dental caries, Incidence

## Abstract

**Background:**

Early childhood caries (ECC) is a serious public health problem in China. Few studies, however, have described the incidence of ECC in China. The purpose of this study was to assess the prevalence and incidence of ECC among preschool children in Wenzhou China.

**Methods:**

Preschool children aged 3–4 years old were surveyed and followed up when they reached 5–6 years of age in the city of Wenzhou in southeast China. The rates of dental caries were determined with prevalence, and incidence density for risk of caries of a person (ID_p_) and of a tooth surface (ID_s_).

**Results:**

The prevalence and decayed, missing, and filled primary teeth (dmft) score of 3–4, 4–5, and 5-6 years old children were 59.8% and 2.9, 71.8% and 4.2, and 76.4% and 4.6, respectively. The ID_p_ was 29.7 and 14.8 persons/100 person-year during the first and second year. The ID_s_ was 5.9 and 2.7 newly affected surfaces/100 surface-year, respectively. The percentage of molars with caries experience increased obviously; the percentage of maxillary central incisors and mandibular incisors with caries experience increased during the first follow-up, whereas it declined during the second follow-up; the others increased gradually.

**Conclusions:**

The prevalence and incidence of dental caries in Wenzhou preschool children were very high with most of the carious teeth left untreated. The molars were the most affected teeth during the observation period.

## Background

Early childhood caries (ECC) is defined as the presence of one or more decayed, missing, or filled surfaces (dmfs) in any primary tooth in children younger than 6 years of age [1]. ECC is a serious public health problem in both developing and developed countries that adversely affects children’s overall health [2]. Studies have shown that the major impact of dental caries on children’s quality of life were irritation, premature tooth-loss, malnutrition, and delayed growth and development [3].

The prevalence of ECC varies from population to population. Peltzer et al. [4] reported that the prevalence of ECC among 3-year-old children from Northern Thailand was 68.5%. Singh et al. [5] stated that the prevalence of ECC among 717 Indian children(3–5 years old of age) was 40%.

In China, dental caries remains a serious public health problem affecting a large proportion of young children. In China, the Third National Oral Health Survey on dental caries showed a prevalence of 66% with a mean number of decayed, missing, and filled primary teeth (dmft) of 3.5 in the 5-year-old age group [6].

Most of the previous studies have described the prevalence of ECC at different chronological ages [7–12]. However, few longitudinal studies have described the incidence of ECC in China [13, 14]. Wong et al. [14] stated that the mean caries increment over the 2 years of the children aged 3–4 years old from Hong Kong was 0.9. Ghazal, T., et al. [15] reported that the two-year incidence of ECC among 1-year-old African-American children in Alabama was 39.3% and the three-year incidence was 65.8%.

Karjalainen et al. [16] stated that the prevalence of dental caries among 3-year-old Finnish children was 8%; then it increased to 28% when they were 6-year-old. The mean dmft among all children was 0.2 at baseline and 0.9 at the follow-up. Grindefjord et al. [17] assessed the prevalence of ECC among 2.5-year-old Swedish children at baseline (11%) and 1-year follow-up (37%). The mean dmfs increment was 1.9.

A longitudinal study on the progression of dental caries in primary teeth was able to find an appropriate access time for ECC prevention [18]. Hence the knowledge on incidence of ECC will help to develop targeted interventions for prevention of ECC, proper planning for the utilization of scare resource available for oral health prevention program and potential decrease in the prevalence of ECC [19].

Despite these and other published studies, further investigation on the prevalence and incidence of ECC in China was necessary. With no organized dental care available for preschool children in Wenzhou, the present study aimed to investigate the prevalence and the incidence of dental caries in primary teeth with increasing age (e.g., from 3–4 to 5–6 years).

## Methods

### Selection of children and sample size

The study site was located in the city of Wenzhou in southeast China, with three districts included. The per capita gross national product of Wenzhou in 2015 was CNY 36,000, making it a moderately developed region of China. The fluoride concentration in drinking water in this area was around 0.2 mg/L.

A stratified cluster sampling method was used to select the study population. Firstly, the whole geographic area was divided into three districts. Secondly, every district was stratified into developed and developing areas based on the economic income. Thirdly, one kindergarten was selected randomly from the developed and developing areas of different districts, respectively. In the end, in the selected 6 kindergartens, three classes were sampled from Grades 1. The children attending Grades 1 are at least 3 years of age and under 4 years of age. In the 2005 survey, the prevalence of ECC was 66% in the 5-year-old age group in China. The sample size estimation showed that the sample size required in this study would be 92 to provide a high precision of the true prevalence with 95% confidence interval of plus or minus 2%. Because the age of participants was less than 5 years old, it was suspected that the corresponding prevalence of dental caries would be less than 66%. Then, larger sample size was required. Furthermore, in order to improve the precision of the incidence, eventually, 693 children aged 3–4 years were recruited in this study.

During the parents meeting at the beginning of the semester, the aim of this study was explained and the details of the study, including relevant risks, compensation, confidentiality, and contact information, in a printed form were sent to all parents or legal guardians. Written informed consents were obtained from them for the dental health examination on their children. Ethical approval for this study was received from the medical ethics committee of Wenzhou Medical University.

### Dental examination and diagnostic criteria

Dental examination appointments were conducted annually for all eligible subjects by one dentist in the same period of the year for two continuous years.

During the examination, the children were seated on a chair in the classroom. In well-lit premises, the children were examined using a World Health Organization (WHO) probe (no. 621) and a dental mirror under natural light. The outcome variables were the number of dmft and dmfs. The dmft and dmfs were used by examiners to evaluate dental caries following the WHO criteria [20]. Dental caries was detected by visual inspection without radiography examination. All examinations were conducted by a trained dentist. To assess the intra-examiner reliability, 10% of the children were reexamined randomly. Kappa statistics was 0.96, 0.98, and 0.96, respectively.

### Statistical analysis

The collected data was entered in Epidata software, version 3.1, and analyzed using SPSS (SPSS, IL, USA). Descriptive statistics was performed. The rates of dental caries were presented as prevalence, crude caries increment and incidence density. The incidence in the present study was calculated as the incidence density for risk of caries of a person (ID_p_) and of a tooth surface (ID_s_) summarized by formula below [18]:

$$ {\mathrm{ID}}_p=\frac{\mathrm{Number}\ \mathrm{of}\ \mathrm{new}\ \mathrm{caries} - \mathrm{affected}\ \mathrm{subjects}}{\mathrm{Total}\ \mathrm{person} - \mathrm{time}\ \mathrm{at}\ \mathrm{risk}\ \mathrm{for}\ \mathrm{having}\ \mathrm{at}\ \mathrm{least}\ \mathrm{one}\ \mathrm{caries}\ \mathrm{lesion}\ \left(\mathrm{person} - \mathrm{year}\right)} $$

$$ {\mathrm{ID}}_s=\frac{\mathrm{Number}\ \mathrm{of}\ \mathrm{new}\ \mathrm{carious}\ \mathrm{s}\mathrm{urface}\mathrm{s}}{\mathrm{Total}\ \mathrm{s}\mathrm{urface} - \mathrm{time}\ \mathrm{at}\ \mathrm{risk}\ \left(\mathrm{surface} - \mathrm{year}\right)} $$

## Results

A total of 693 children participated in the study. Of these, 87 (12.6%) children who had participated in dental examination only once were excluded. Consequently, only 606 children were included in the present study. Of these, 269(boys 140, girls 129) were from developing area, and 335(boys 175, girls 160) were from developed area. The mean age of the children was 3.4 years old; 278 (47.9%) were girls, and 328 (52.1%) were boys. 606 children accepted dental examination at least twice, of which 22 (3.6%) children attended the first and third examinations. Therefore, the data of 22 children was used only for the prevalence analysis. The remaining 584 (96.4%) participated in incidence density analysis (Table 1).

**Table 1:** Distribution of children at the time of examination

Examination time			Children attending examination		
2011	2012	2013	*N*	%	Data analysis
√	√	√	377	62.2	Incidence density and prevalence
√	√		46	7.6	Incidence density and prevalence
√		√	22	3.6	Prevalence
	√	√	161	26.6	Incidence density and prevalence
		Total	606	100.0	

ECC was first observed in children at 3–4 years of age. The prevalence of 3–4, 4–5, and 5–6 years old children were 59.8%, 71.8%, and 76.4%, respectively. The mean dmft score of 3–4, 4–5, and 5–6 years old children were 2.9 (standard deviation [21] = 3.7), 4.2 (SD = 4.4), and 4.6 (SD = 4.5), respectively; the dmfs scores were 4.9 (SD = 9.0), 9.4 (SD = 12.4), and 11.5 (SD = 13.7), respectively (Table 2). The crude caries increment during the first follow-up was 1.1 (SD = 1.7), whereas it was 0.7 (SD = 1.1) during the second follow-up. The percentage of untreated carious teeth in 5–6-year-old children in this study was 83.6%. The incidence of caries-affected persons (ID_p_) observed during the first follow-up was 29.7 person/100 person-years, whereas it dropped to 14.8 person/100 person-years during the second follow-up. The rate of new caries-affected tooth surface (ID_s_) during the first follow-up was 5.9 surfaces/100 surface-years, whereas it decreased to 2.7 surfaces/100 surface-years during the second follow-up (Table 3).

**Table 2:** Prevalence of dental caries during 2011–2013

Examination time	N	Age	ECC	Prevalence (%)	dmft	dmfs
2011	445	3.6	266	59.8	2.9	4.9
2012	584	4.6	419	71.8	4.2	9.4
2013	560	5.6	428	76.4	4.6	11.5

**Table 3:** Frequency, incidence, and rates of risk of caries-affected children/surfaces

	Observation period (years)	
	2011–2012	2012–2013
*N* (persons)	423	538
Number of new caries-affected children	51	22
Person-years of observation	172	149
ID_p_/100 persons at risk	29.7	14.8
*N* (surfaces)	35464	47344
Number of new caries-affected surface	1967	1130
Surface-years of observation	33374	42309
ID_s_/100 surface at risk	5.9	2.7

**Fig. 1:**
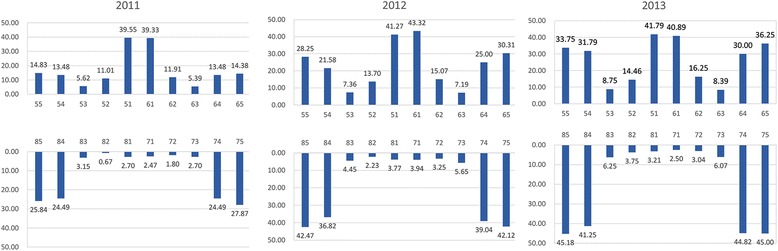
The prevalence of dental caries by tooth position during 2011–2013

Figure 1 showed the prevalence of dental caries by tooth type. The percentage of maxillary central incisors with caries experience increased from 39.3% to 43.3% during the first follow-up, whereas it declined to 40.9% during the second follow-up. In maxillary, the percentage of teeth have caries experience increased from 11.9% to 16.3% in lateral incisors, 5.4% to 8.4% in canines, 13.5% to 30.0% in 1^st^ molars and 14.4% to 36.3% in 2^st^ molars during the two-year follow-up. The percentage of mandibular incisors with caries experience increased during the first follow-up, whereas it declined during the second follow-up. In mandibular, the percentage of teeth have caries experience increased from 2.7% to 6.1% in canines, 24.5% to 44.8% in 1^st^ molars and 27.9% to 45.0% in 2^st^ molars during the two-year follow-up.

## Discussion

The study found a high prevalence of dental caries at 4–5 years old of age in Wenzhou, which was higher than the results from the national survey [6]. Wenzhou is a moderately developed region in China. With economic development, children have more access to cariogenic sugary food and drinks; however, oral health knowledge, attitudes, and behaviors are lacking. This resulted in a higher prevalence than the national level.

The present study showed that the prevalence of ECC rose sharply from 59.8% to 71.8% during the first follow-up; after that, it increased to 76.4% during the second follow-up. Meanwhile, the percentage of untreated carious teeth in 5–6-year-old children in this study was 83.6%. The high level of untreated caries could be ascribed to the low pediatric dentist/population ratio in Wenzhou, attitude of parents toward the primary dentition, and high cost of treatment.

Among children unaffected by caries at 3–4 years, 29.7% were affected during the first follow-up; the children unaffected by caries at 4–5 years, 14.8% were affected during the second follow-up. The crude caries increment during the first follow-up period was 1.1; it was 0.7 during the second follow-up period. It showed that the rate of caries increment became lower in the second follow-up. Biologically, this might be due to the higher susceptibility of newly-erupted teeth to dental caries because of the colonization by microorganisms. Weintraub et al. [22] assessed the development of ECC among 126 caries-free children aged 6–44 months at baseline who did not receive fluoride varnish treatment. Including non-cavitated lesions, 29% had caries incidence after 1-year; at the 2-year follow-up, 24% of children who were caries-free at 1-year follow-up had caries. The incidence of caries-affected persons (ID_p_) observed during the first follow-up was 29.7 person/100 person-years. Within one year, a rapid destruction of tooth was observed. It is of significant interest and needs further investigation to identify important associated factors in this rapid caries progression. Among previous studies, the factors found to be associated with ECC have included low socioeconomic statue, high consumption of sugar snacks or beverages, poor diet, irregular feeding practices, nutritional problems, poor oral hygiene, and higher levels of Mutans streptococci [13, 23–26].

The analysis of variation in the prevalence of individual tooth revealed that during the follow-up period, the molars were mostly affected, while the incisors had been affected mostly before the survey. During the second follow-up, a little decline was observed in the percentage of carious incisors due to the beginning of mixed dentition. With time, the caries rate of other teeth increased gradually. Previous studies revealed that susceptibility to caries in the study children occurred in the first 3–6 months after the teeth had erupted into the oral cavity [18]. A preventive program must be implemented early in life. Caries affects the maxillary primary incisors and first primary molars in a way that reflects the pattern of eruption [27]. The longer a tooth is exposed to the caries challenge, the more the chances that it will be affected.

Dental caries is a preventable disease, and it can be stopped and even potentially reversed during its early stages. The present study indicated a high caries rate during the follow-up period. If appropriate prevention programs are implemented, the result will be fruitful. A cohort study is suitable for calculating the incidence rate and better characterizing the incremental rate of disease events. The results are easily interpreted. In this study, the incidence of caries-affected persons (ID_p_) observed during the first 1-year follow-up period was 29.7 person/100 person-years, which indicated that if 1000 persons are followed up for 1 year, 297 persons are expected to become carious.

The present study had several limitations. Selected bias was possible. The development of ECC in children younger than 3 years old is unclear. Further investigation is required to identify the risk factors for ECC development. In conclusion, the rate of caries in the study children was extremely high. The molars were the most affected teeth during the observation period, indicating that the access time for ECC prevention should be earlier.

## Conclusions

The prevalence and incidence of dental caries in Wenzhou preschool children were very high with most of the carious teeth left untreated. The molars were the most affected teeth during the observation period.

## Bullet points

### Why this paper is important to pediatric dentists

The prevalence of ECC varies from population to population, this study provide the incidence of ECC in China. This paper helps to clearly understand the rate of caries progression of each individual tooth over time to find an appropriate access time for ECC prevention.

## Abbreviations

dmfs: Decayed, missing, and filled surfaces
dmft: Decayed, missing, and filled teeth
ECC: Early childhood caries
SD: Standard deviation
WHO: World Health Organization.
